# A Survey of Deep Learning-Based Source Image Forensics

**DOI:** 10.3390/jimaging6030009

**Published:** 2020-03-04

**Authors:** Pengpeng Yang, Daniele Baracchi, Rongrong Ni, Yao Zhao, Fabrizio Argenti, Alessandro Piva

**Affiliations:** 1 Institute of Information Science, Beijing Jiaotong University, Beijing 100044, China; 14120339@bjtu.edu.cn (P.Y.); yzhao@bjtu.edu.cn (Y.Z.); 2 Beijing Key Laboratory of Advanced Information Science and Network Technology, Beijing Jiaotong University, Beijing 100044, China; 3 Department of Information Engineering, University of Florence, Via di S. Marta, 3, 50139 Florence, Italy; daniele.baracchi@unifi.it (D.B.); fabrizio.argenti@unifi.it (F.A.)

**Keywords:** image forensics, multimedia forensics, source identification, data driven methods

## Abstract

Image source forensics is widely considered as one of the most effective ways to verify in a blind way digital image authenticity and integrity. In the last few years, many researchers have applied data-driven approaches to this task, inspired by the excellent performance obtained by those techniques on computer vision problems. In this survey, we present the most important data-driven algorithms that deal with the problem of image source forensics. To make order in this vast field, we have divided the area in five sub-topics: source camera identification, recaptured image forensic, computer graphics (CG) image forensic, GAN-generated image detection, and source social network identification. Moreover, we have included the works on anti-forensics and counter anti-forensics. For each of these tasks, we have highlighted advantages and limitations of the methods currently proposed in this promising and rich research field.

## 1. Introduction

With the development of modern techniques, digital imaging has become an important component in our daily life. It is easy for us to capture digital images with devices such as smartphones and digital reflex cameras, embellish them by using photo-editing software, and then upload them to social network platforms to share the important moments of our life with our friends. The ease with which we handle digital images, however, is a double-edged sword. Forged images are becoming more and more widespread in our life and seeing is no longer believing [1,2], especially with the advent of techniques based on artificial intelligence (AI) such as generative adversarial networks (GAN) [3] that can be exploited by malicious actors to spread “fake news” [4].

**Figure 1:**
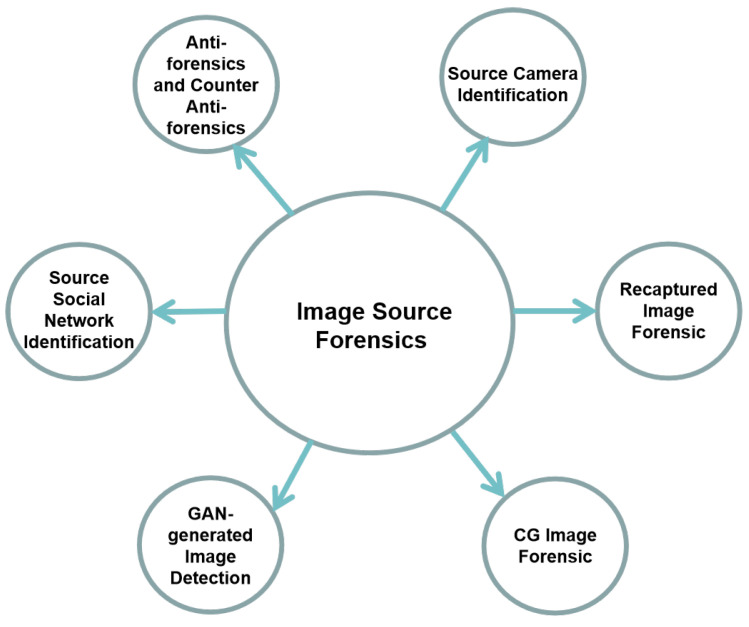
The structure of the presented review.

In order to verify the authenticity and integrity of a digital image, a number of techniques, known collectively as “digital image forensics” [5], were developed during the last years. Within this research area, source image forensics tries to answer the general question “where is this digital image from?”, and to do so multiple sub-topics can be explored, represented in Figure 1: source camera identification, recaptured image forensic, computer graphics (CG) image forensic, GAN-generated image detection, and source social network identification.

In the past the task of source image forensics has been dealt with several algorithms based on statistical analysis and pattern recognition. More recently, improvements in computing capabilities sparked a renewed interest in techniques based on machine learning. In particular, deep learning-based schemes have been successfully applied in the field of source image forensics, and have proved their effectiveness in various competitions [6,7].

Techniques based on artificial neural networks have been known for many years and have gone by many names. A series of breakthroughs in 2006 [8,9,10], however, made the use of deep neural networks viable and gave rise to the field known as deep learning [11,12]. After that, methods based on deep learning have consistently achieved remarkable results on a series of tasks such as handwritten digital recognition and image classification, often beating competing approaches based on conventional schemes. A number of specialized techniques have been developed in the field of computer vision, including convolutional neural networks (CNN), recurrent neural networks (RNN), and generative adversarial networks (GAN). Among them, CNNs have been shown to be effective when dealing with image-related tasks, and have been subsequently adopted as a basis for numerous digital image forensics methods.

Basic components of CNNs consist mainly of convolutional layers, pooling layers, and activation functions, which are stacked together to construct the architecture of CNNs. According to Khan et al. [13], recent innovations in CNNs building and training can be categorized as structural reformulation, parameters optimizations, regularization, loss function. Among those, structural reformulation plays the most important role in improving the performance, and can be divided into seven different classes: spatial exploitation, depth, multi-path, width, channel boosting, feature map exploitation, and attention based CNNs. Typical CNN architectures such as AlexNet [14], VGGNet [15], GoogleNet [16], ResNet [17], DenseNet [18], Xception [19], SENet [20], Siamese Network [21], are well known. We refer the interested reader to the review by Khan et al. [13], Gu et al. [22] for further details about deep learning.

**Figure 2:**
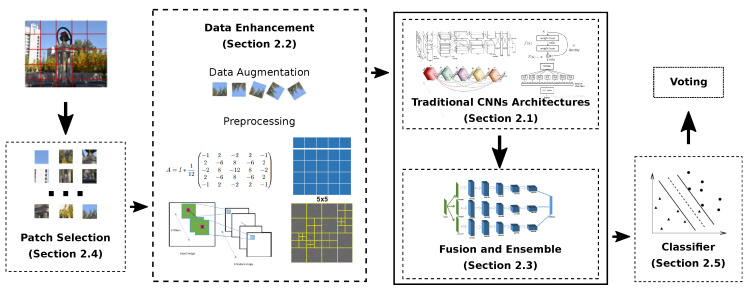
The framework of the deep learning-based algorithms for source forensics. (Section X.X) indicates the subsection where the related technique is described in detail.

The impressive results obtained by deep learning-based methods in image source forensics motivate us to provide a comprehensive review of those approaches in such a way as to allow a neophyte to come into this field with some help. In this survey paper, we did our best to collect all the related paper which have been published in journals, conferences, and arXiv. We observed that all of them have some common modules, and we propose a unifying framework where all these schemes fit, thus simplifying their comparison. The framework is visually represented in Figure 2. First, the input, a full resolution image, is usually cropped into small and fixed-size pixel patches. Then, all patches are processed, or a patch selection strategy can be applied to choose the patches that are more useful for the following task. Next, these patches can be pre-processed by a spatial filter to improve their signal-to-noise ratio (SNR). After that, they are fed to a convolutional neural network (CNN). Classification of a single patch can be achieved either by having a softmax layer at the end of the network, or by training a separate classifier on the features extracted by the last layer of the CNN. In the end, the classification result for the original image can be obtained by voting among the pixel patches. It should be noted that our general framework includes all the ideas used in the methods that we have reviewed; until now, however, no single method is using all those techniques. In Figure 2, optional parts are denoted by a dotted contour. Starting from the general framework, we have divided the reviewed algorithms in the following five separate sub-sections, according to their main contributions:

1. adoption of traditional convolutional neural networks (T.CNN) for source camera identification tasks;
2. improvement of performance by using data enhancement (D.A.), including data augmentation and data preprocessing;
3. improvement of performance through fusion and ensemble (F./E.);
4. improvement of performance by means of patch selection (P.S.);
5. adoption of different classifiers (C.).

The most significant network parameters of the reviewed works are summarized in Table 1 and Table 2. Then, in Table 3 and Table 4, we have summarized the experimental settings and the performance achieved by those architectures, as well as by some conventional CNNs.

The remaining part of this review is organized as follows. First of all, in Section 2 we will describe the deep learning-based methods that deal with the most important topic of this area, i.e., source camera identification. Then, in Section 3, Section 4, Section 5, Section 6 we will present an overview on the methods dealing with the remaining sub-topics: recaptured image forensic, CG image forensic, GAN-generated image detection, and source social network identification, respectively. In Section 7 we will describe anti-forensics and counter-anti-forensics algorithms based on deep learning methods. A description of the results obtained by the described techniques will be given in Section 8. Finally, in Section 9, we will provide our analysis and conclusion on deep learning based source image forensics.

## 2. Source Camera Identification

One of the hot topics in multimedia forensics is source camera identification, the purpose of which is to trace where an image is from. Identifying the source camera is an important step in pointing out the owner of illicit images (e.g., crime scenes, terroristic act scenes, etc.) and ensuring the security and trustworthiness of such digital data.

### 2.1. Traditional Convolutional Neural Networks (T.CNN)

Early works focused on applying to the problem of source camera identification traditional convolutional neural networks consisting of stacked convolutional layers. To the best of our knowledge, deep learning-based schemes for source camera identification were firstly introduced by Bondi et al. [23]. This path-breaking method used a simple architecture with five layers, including three convolutional steps and two fully connected layers, working on 48×48 patches. The authors tested their network for both instance- and model-level camera identification, obtaining accuracies of 29.8% and 72.9%, respectively. Moreover, model-level camera identification increased to 94.1% on full resolution images with a voting strategy on the respective image patches. Focusing on images captured by mobile phones, which nowadays are the most popular image acquisition devices, Freire-Obregón et al. [24] proposed a six-layer CNN architecture, including two convolutional layers, one max pooling layer and three fully connected layers. The activation function used in this work was the Leaky Rectified Linear Unit (L-ReLU) which, as reported by the authors, led to slightly better performance than those obtained by using ReLU activations. Then, Huang et al. [25] presented an architecture similar to the one proposed by Bondi et al. [23], and the authors were able to improve over the accuracy obtained by Freire-Obregón et al. [24] by using Batch Normalization and more convolutional layers. Following along the line of deeper CNN architectures, Yao et al. [26] (code available at https://github.com/grasses/Camera-Identification) put forward a 13-layer convolutional neural network. The proposed method is robust against JPEG compression and noise adding; however, it is not resistant to re-scaling operation.

Chen et al. [27] investigated the use of a residual neural network (ResNet) with 26 layers for source camera identification, and proved its effectiveness with multiple experiments: the accuracies obtained for brand-, model-, and device-level identification were 99.12%, 94.73%, and 45.81%, respectively. According to their paper, ResNet has better performance than AlexNet, GoogleNet, and the scheme from Bondi et al. [23]. Ding et al. [28] extended this last method by combining ResNet architecture with a multi-task learning strategy, further improving the performance. The three tasks (brand-level, model-level, and sensor-level classification) are integrated into one framework and share the weights of the CNN architecture.

Several works [29,30,31,32,33] applied to source camera identification new architectures from computer vision such as DenseNet, XceptionNet. Marra et al. [29] used XceptionNet to obtain overall accuracies of 95.15% for pixel patches and 99.31% for the full resolution image by using a voting strategy.

All the works cited so far are intended to solve source camera identification in a closed-set scenario, where there is the assumption that we have a perfect knowledge of all the devices that could possibly capture the query images, which means that the acquisition devices for test data are the same used for acquiring training data. A more realistic case, however, is the open-set scenario, where information about query images is not completely known. Recently, a number of deep learning methods for this more challenging scenario have been proposed. Bayar and Stamm [34] presented two different schemes to address the open-set problem for camera model identification, which aims to judge whether the device that captured the query image is known or unknown. The first one uses, in place of a classification layer, a confidence score mapping with a thresholding strategy to evaluate whether the true source camera model is known or unknown. The other approach uses a different classifier on features extracted by a CNN. Mayer and Stamm [35] tried to measure similarity among images by using a siamese network. Features are extracted from the last layer of a CNN and fed into a siamese network to learn a measurement of source similarity, which allows for verifying if two query images are captured by same device or not.

It should be noted that the evolution of CNN architectures proposed for Source Camera Identification tasks closely follow the one of architectures proposed for computer vision tasks. This is somewhat to be expected as CNNs are well-suited for that kind of tasks, and thus a great deal of research on neural architectures produced by the community was focused on solving those problems. Given the encouraging results obtained so far, it is logical to assume that the forensics community will keep on building on new CV architectures.

### 2.2. Data Enhancement (D.E.)

Data enhancement, including data augmentation and pre-processing, has been widely used as an effective way to improve the performance when dealing with computer vision tasks. These techniques have also been adopted for source camera identification schemes. Bondi et al. [36,37] normalized the images by subtracting the pixel-wise mean value, which is a popular way to center the data and helps the network to learn faster. Kamal et al. [31] applied five different data augmentation operations: random crops, random rotations, image manipulations (including JPEG compression, gamma correlation, and resizing), images addition, and empirical mode decomposition. It should be noted that the authors increased the training set size by collecting more images from Flickr (https://www.flickr.com/). The results demonstrated that adding more images has a great impact to performance: considering that deep learning-based methods are data-driven, increasing the number of training examples definitely leads to higher detection accuracies. Team GPU_muscle [30,32] collected more than 500 GiB of photos from various resources (Flickr, Yandex.Fotki, Wikipedia Commons, mobile reviews, and others) and obtained an accuracy of more than 98% by training traditional CNNs. In addition, using manipulated images such as enhanced images with gamma correction, JPEG compressed images, and images transformed by resampling operation, to some extent, enhances the robustness of the CNN model.

Preprocessing techniques to improve the signal-to-noise ratio (SNR) of input data have been introduced following the intuition that the main difference between computer vision and image source identification tasks lies in the importance of image contents. Usually, computer vision tasks are seriously dependent on the image contents, whereas the opposite is true when dealing with source camera identification. In the latter case, the correct class to be attributed to an example is heavily dependent on the noise component introduced by camera acquisition. Based on this observation, some researches [38,39,40,41,42] proposed to reduce the interference of the image contents using two kinds of preprocessing. The first technique is based on the idea of applying a denoising filter *F* to the input image *I*, and then subtracting the result of that operation from *I* itself, thus obtaining the noise *N*:

(1) N=I-F(I).

Tuama et al. [39] chose a wavelet-based denoiser, as filters of that kind have been widely used in model-based schemes based on Photo Response Non Uniformity (PRNU) for source camera identification. Bayar and Stamm [38] evaluated the effect of a median filter with 3×3 windows (MFR). The second preprocessing technique is based on the idea that the noise can be easily obtained by using a spatial filter *G*:

(2) N=I∗G

Tuama et al. [39] also tested the effectiveness of an high-pass (HP) filter. According to that work, HP filters yield better results than wavelet-based denoiser when used in CNN-based schemes. Ding et al. [28] evaluated the case of gaussian filter residuals with three scales (3×3, 5×5, 7×7) and verified their effectiveness.

Following the idea of spatial filters, Yang et al. [42,43] presented self-learning filters as a way to further improve the SNR. Self-learning filters can be trained together with the rest of the network. Inspired by SPAM features [44], Bayar and Stamm [45] (code available at https://gitlab.com/MISLgit/constrained-conv-TIFS2018) proposed a novel constrained convolution which ensures that learned high-pass filters are within a given bound. In particular, the central weight of the convolutional kernel is set to -1 and the sum of the other weights is equal to 1. Instead of designing the filter, Wang et al. [41] used local binary patterns (LBP) to code the image. The images are first processed by LBP coding operation in the preprocessing step, and then fed into CNN architecture. Self-learning filters, constrained convolutions, and LBP coding are reported to outperform high-pass filters.

Recently, Zuo [40] evaluated how the performance of CNN models are impacted by the use of two preprocessing techniques: laplacian gaussian smoothing filters and non-local means denoising filters. Results indicate that the CNN model without pre-procession provides better performances. The author gave a possible explanation from perspective of the training data and strategy. It should be noted, however, that the dataset used was only composed of images captured by three camera models, and that the number of epochs during training phase was small due to limitations in the available computing power. For these reasons, extended experiments should be conducted to verify the effect of preprocessing in future.

The analysis of data enhancement techniques used in source camera identification tasks highlight an important difference with respect to classical DE methods used in machine learning. Commonly used augmentation algorithms used by the ML community focus on helping the network become rotation- or scale-invariant by presenting image contents in different conditions. In source camera identification tasks, on the contrary, most DE technique aims to reduce the influence of image contents by filtering out information deemed not useful. As those two classes of enhancement methods are not mutually exclusive, future works could attempt to combine them to come up with a more comprehensive way to make the network more robust.

### 2.3. Fusion and Ensemble (F./E.)

Fusion and ensemble strategies aim to enhance performance by fusing multiple models and features together. Yang et al. [42] constructed a content-adaptive fusion network by merging three models together, thus significantly increasing the overall accuracy with respect to the single models. Bayar and Stamm [38] combined constrained convolutions and MFR at the first layer of the CNN architecture and obtained a slight improvement over a constrained CNN. Kamal et al. [31] used the ensemble feature of DenseNet201 trained using three image scales (64×64, 128×128, 256×256), which is beneficial to the CNN model. Ferreira et al. [33] proposed to integrate InceptionNet and XceptionNet architectures to boost performance.

### 2.4. Patch Selection (P.S.)

It is well known that the PRNU noise that can be extracted from an image is related with its contents. Smooth, non-saturated areas with high luminance are good for PRNU estimation. Furthermore, content-adaptive processes such as CFA demosaicing and JPEG compression can be applied during image acquisition. Therefore, choosing different areas in the image could have some effect on the performance. Based on this fact, a good strategy for choosing the best pixel patches to be used for CNN training can be essential to obtain higher performance. Bondi et al. [36] (code available at https://github.com/polimi-ispl/camera-model-identification-with-cnn) only select for training the pixel patches whose average values are close to half of the image dynamic range. Another criterion for patch selection, which aims to find the better textured pixel patches with the half of the image dynamic, was proposed by Kamal et al. [31], Bondi et al. [37]. In this approach the quality value for a pixel patch is computed from its variance and mean. Pixel patches with higher measure value are used to train the CNN model. Güera et al. [46] proposed a CNN-based solution to estimate, for each pixel patch, a value representing the camera-model-attribution reliability. Yang et al. [43] adopted a different approach where pixel patches were separated into three subsets according to their mean and variance. Then, a different CNN model would be trained on each subset. Finally, query pixel patches would be classified using the model corresponding to their characteristics.

It appears that, currently, the dynamic range of a patch is considered to be the best descriptor for its usefulness for the task at hand. Experimental results confirm that such an approach is sensible. It would however be interesting to explore more diverse descriptor, perhaps taking into account the peculiarities of the neural network that will be subsequently used to classify the selected patches.

### 2.5. Classifier (C.)

Some computer vision works [47,48,49] claim that using a separate SVM classifier on the features learned by a CNN instead of a softmax layer can improve classification performance. In the same way, some image forensics researchers have recently explored if the adoption of different classifiers can improve the performance. In particular, Huang et al. [25], Kamal et al. [31], Bondi et al. [36,37] and Bayar and Stamm [34,38] proposed two-stage learning strategies by feeding the features extracted by a CNN model into a different classifier such as a Support Vector Machine (SVM), Extremely Randomized Trees (ERT), cosine similarity measure, nearest mean score, and deep learning architecture with squeeze and excitation block. The results confirm that these classifiers can achieve better performance with respect to simple softmax layers.

### 2.6. Summary

The most significant network parameters and the experimental settings of the reviewed works are summarized in Table 1, where we have identified twelve main architectures (using the short name A1, A2, …, A12). Then, in Table 3 we have summarized the experimental settings and the performance achieved by those architectures, as well as by some conventional CNNs.

## 3. Recaptured Image Forensic

Recaptured image forensic deals with the task of establishing whether an image has been recaptured or not, that is if it has been generated by capturing a printed picture or a screen display with an acquisition device. Yang et al. [50] presented an effective and practical deep learning-based method to address this problem: Laplacian Convolutional Neural Networks (L-CNN). In this technique the Laplacian filter is embedded into the first layer of a CNN to improve the noise signal ratio introduced by recapture operations. In that paper five different kinds of high-pass filters have been evaluated. Experimental results showed that performance obtained using laplacian filter is better than the one obtained by using other high-pass filters, or by not using filters at all. According to the paper, L-CNNs achieve 96% detection accuracy even when applied to 64×64 image patches. To the best of our knowledge, this is the first work based on deep learning to detect recaptured images. Choi et al. [51] tested a nine-layer CNN on 64×64 patches and obtained a detection result on the original images by using a voting strategy. The authors report slightly improved results over model-based methods. Li et al. [52] proposed a new framework by combining CNNs with RNNs. Instead of using a Laplacian filter in the first layer, the authors considered the convolutional operation as the preprocessing. The weights of the convolutional operator can be automatically learned during the training phase. Features extracted from trained CNN model were then fed into a recurrent neural network to classify the images. Aforementioned algorithms were evaluated on a small-scale dataset. Recently, Agarwal et al. [53] developed a diverse large-scale dataset for evaluating recaptured image forensic techniques. The dataset consists of 14,500 recaptured images and 14,500 original images. Those images were captured by various devices such as cameras, displays, scanners, printers. The authors also proposed an eight-layer CNN with 16 different kinds of gaussian filtering residuals in the first layer. The reported detection accuracy was up to 99.9% for 64×64 pixels patches, which is a great improvement over model-based schemes.

The most significant network parameters and the experimental settings of the reviewed works are summarized in Table 2, where we have identified four main architectures (using the short name B1, B2, …, B4). Then, in Table 4 we have summarized the experimental settings and the performance achieved by those architectures, as well as by some conventional CNNs.

We can notice how most of the techniques proposed for recaptured image forensics are base on some kind of manual of automatic filtering. As an image’s subject is not useful for establishing whether the image has been recaptured, those filters attempts to discard that information while, at the same time, highlighting the traces left by the recapturing operation.

## 4. Computer Graphics Image Forensic

Another possible source of a digital image is represented by Computer Graphics algorithms, so proper methods to detect this kind of origin have also been developed. In this field, Yu et al. [54] evaluated VGG-based architectures for CG image detection and found that their performance could be improved by dropping max-pooling layers. The authors explained that pooling layers could lead to a loss of association between adjacent pixels. Therefore, they presented a six-layer CNN without any pooling layer and achieved detection accuracies of over 98% on 32×32 patches. He [70] tested several training strategies for VGG-19 and ResNet-50 architectures. Specifically, transfer learning technique was applied during the training phase and a fine-tuned ResNet-50 model was found to have the best performance. The authors report an average detection accuracy of about 96.1% on DSTok dataset. Building on the hybrid CNN-RNN approach of Li et al. [52], He et al. [55] presented a similar framework with a dual-path CNN to identify CG images. In this approach, 96×96 patches are firstly converted to the YCbCr color space. Then, the luminance component is processed by a Schmidt filter bank to generate 13 different kinds of filtered responses. Lastly, the pair of chrominance components Cb,Cr and the filtered responses of the luminance component are separately fed into a four-layer CNN. Using this technique, the authors improved over the results obtained by Yu et al. [54] by 4 percentage points. Yao et al. [56] designed a five-layer CNN where the inputs are preprocessed by using high-pass filters. In this work, the authors explored three high-pass filters that were first introduced in the field of steganalysis: SQUARE5x5, SQUARE3x3, and EDGE3x3. Cui et al. [72] evaluated the use of ResNet-50 using the PRNU noise as input, and their architecture achieved a detection accuracy of 98% on Columbia Photographic Images and Photorealistic Computer Graphics Dataset. Instead of using fixed filters in the preprocessing step, Quan et al. [57] (code available at https://github.com/weizequan/NIvsCG) proposed a CNN with 32 convolutional operations in the first layer so that the weights of convolutional operator can be learned during the training phase. The results reported in their paper show that the performance of this method is better than the conventional methods, like Geo [77], SPAM [44], and Mfra [78], which indicate the effectiveness of CNN-based method on computer graphics (CG) image forensic. Rahmouni et al. [58] (code available at https://github.com/NicoRahm/CGvsPhoto) presented a novel statistical features extraction (SFE) layer and embed it between the last convolutional layer and the first fully connected layer. The SFE layer would extract four features: mean, variance, maximum, and minimum. The authors also explored feeding those features into different classifiers such as LDA and SVM. According to their paper, the best results are obtained by the CNN model trained in an end-to-end way. Continuing on this path, Nguyen et al. [73] improved detection performance by using a more powerful feature extractor: VGG-19. In this approach, the outputs of the convolutional operations before first three max pooling layers were extracted and the final features were calculated by computing their mean and variance. Then, three groups of those final features were fed into a fused 1-D CNN with two convolutional and three fully connected layers. Sharing a similar idea with the last two methods, De Rezende et al. [74] explored using other feature extractors and different classifiers: softmax, k-nearest neighbors, XGBoost, and SVM. In the end, ResNet-50 was chosen and the outputs of its 49th layer were used as features. The authors report that combining ResNet50 with a SVM classifier with RBF kernel achieved the best performance.

The most significant network parameters and the experimental settings of the reviewed works are summarized in Table 2, where we have identified five main architectures (using the short name C1, C2, …, C5). Then, in Table 4 we have summarized the experimental settings and the performance achieved by those architectures, as well as by some conventional CNNs.

It can be noted that many recent works adopt approaches based on the extraction of statistical features from filtered images. All of them, however, only compute simple indicators such as mean and variance. It would be interesting to explore the possibility of using more sophisticated statistical features and whether those variant could yield an improvement on photo-realistic CG images.

## 5. GAN-Generated Image Detection

In recent years, a number of deep learning techniques capable of generating fake multimedia contents has been developed. Those methods, collectively called “deepfakes”, include autoencoders (AE) and generative adversarial networks (GAN). This pose significant challenges to the forensics community, as the contents generated by those techniques are much more realistic than the ones generated by computer graphics algorithms. Numerous researches and competitions [79] have focus on the detection of deepfake multimedia, such as the MFC2018 and DFDC launched by NIST and Facebook, respectively, and some surveys on this topic have been published. Verdoliva [80] presented an overview of media forensics and deepfakes. Nguyen et al. [81] came up with a survey of algorithms used to create deepfakes and, more importantly, methods to detect deepfakes proposed in the literature to date. Tolosana et al. [79] provided a thorough review of techniques for manipulating face images including DeepFake methods, and methods to detect such manipulations.

The most recent topic related to source image forensics is the detection of content generated by means of GANs. Here, Marra et al. [82] evaluated the performance of several image forensic detectors and popular computer vision CNN architectures on GAN-generated images detection. More specifically, the authors used four image forensic detectors: the method proposed by Fridrich and Kodovsky [83], the one by Cozzolino et al. [84], the one by Bayar and Stamm [85], and the one by Rahmouni et al. [58]. The reviewed CNN architectures were: DenseNet [18], InceptionNet v3 [86], XceptionNet [19], and the Cycle-GAN [87] discriminator. Experimental results showed that XceptionNet has the highest average detection accuracy even for images that have undergone Twitter-like compression. Haodong et al. [88] reported on an experimental investigation about the effectiveness of forensic detectors for GAN generated image detection, and in particular fake face images generated by Deep Convolutional Generative Adversarial Networks (DCGAN) [89] and Wasserstein Adversarial Networks (WGAN) [90]. Four approaches were evaluated in this work: GAN discriminator, face quality assessment, Inception score, and VGG-features with FLD. The best performance was obtained by using VGG-features with FLD. However, its generalization performances are limited when test set images are generated by different GAN schemes than the one used for training. Focusing on the differences in color composition between original and GAN generated images, Li et al. [91] presented a method where a feature set based on co-occurrences matrices is used to capture color image statistics. Firstly, the color image is transformed to RGB, HSV, and YCbCr spaces. Then, residual images would be generated for RGB, H, S, Cb, Cr channels, and co-occurrence matrices for all the residual images would be calculated. The extracted features would be finally fed into a binary classifier. The proposed method was evaluated on three public faces datasets (celebA [92], HQ-CelebA [93], LFW [94]) with four kinds of generated images (deep feature consistent variational auto-encoder (DFC-VAE) [95], DCGAN, WGAN-Gradient penalty (WGAN-GP) [96], Progressive Growing Generative Adversarial Networks (PGGAN) [93]), and it was able to obtain better performance than the one of model-based texture feature set [97]. McCloskey and Albright [98] extracted features from color and saturation space to detect PG-GAN generated image. On one hand, the standard rg chromaticity space is applied and the bivariate histograms of r, g components are fed into a INH network [99]. On the other hand, two groups of saturation measurements are extracted as features and SVM is used to classify PG-GAN generated images. The dataset produced in conjunction with the US National Institute of Standards and Technology’s Media Forensics Challenge 2018 was used to evaluate the performance of those two schemes. According to the report, saturation statistics provided better performance. Mo et al. [59] expected that the main difference between the original and GAN-generated images would be reflected on the residual domain. Therefore, they presented a three-layer CNN with a Laplacian filter preprocessing to identify fake face images generated by PG-GAN. From the perspective of artificial fingerprints, Marra et al. [100] explored a PRNU-based scheme for GAN-generated image detection. Three GAN architectures are considered in this work: Cycle-GAN, Pro-GAN, and Star-GAN. The results demonstrated that those GAN schemes would leave artificial fingerprints into the generated images.

The most significant network parameters and the experimental results of the reviewed works are summarized in Table 2, and in Table 5.

Based on our review of GAN-generated image detection works, we can broadly classify the proposed methods in three categories: existing detection methods; approaches based on the analysis of color characteristics of the images; and techniques based on the analysis of images’ residual/noise. However, GANs are a very popular topic among machine learning researchers, and thus we expect GAN-generated images to become increasingly difficult to be detected. Existing techniques are likely to become obsolete in a short time, and for this reason forensics researchers will need to keep building new, effective detection methods.

## 6. Source Social Networks Identification

In recent years, social networks such as Facebook, Google+, and Twitter became more and more important in the daily life of a large part of the world population. According to Caldelli et al. [75], on average 350 million photo are uploaded daily on Facebook and around 60 millions monthly on Flickr. To the best of our knowledge, Amerini et al. [60] were the firsts to propose a CNN-based algorithm to identify from which social network a query image has been downloaded. The authors indicated that image manipulations applied by social networks usually include compressing as JPEG and resizing the original file, and that different social networks use different parameters for those operations. Therefore, it is possible to identify the source social network by looking at the discrete cosine transform (DCT) coefficients of the resulting image. Inspired by the work of Wang and Zhang [101], the authors extracted from each image the histogram of the first nine DCT coefficients and fed them into a simple 4-layer 1D CNN, obtaining great performance on source social network identification. Caldelli et al. [61] presented another scheme based on a 2D CNN with a preprocessing step. In the pre-processing stage, the PRNU noise of the image would be extracted. This noise would be then fed into a 6-layer 2D CNN. This technique obtains performance comparable with the one from the first method.

The most significant network parameters and the experimental settings of the reviewed works are summarized in Table 2, where we have identified the two main architectures as E1 and E2. Then, in Table 4 we have summarized the experimental settings and the achieved performance.

The works presented in this section assume that images published on a social network will be re-encoded as JPEG after being processed by some unknown set of operations. Even if this is a sensible assumption, there is no guarantee that the specific processing pipeline used by a social network will stay the same over time. A simple software update on the backend software of a social platform could dramatically change how the images are processed, thus making an existing classifier obsolete. For this reason, all the methods described in this section require a constant update where social-network-processed images are continuously used to retrain the models. This is, however, inevitable due to the lack of knowledge about the inner working of social platforms.

## 7. Anti-Forensics and Counter Anti-Forensics

Image anti-forensics are techniques that aim to make forensics algorithms fail by modifying the images in a visually imperceptible way. Their goal is to prompt the image forensics community to come up with more robust forensics schemes. Recent developments in deep learning research led to the development of generative adversarial networks (GANs), novel techniques that proved to be very effective in deceiving many existing image forensics approaches. Güera et al. [102] trained a DenseNet-40 model for source camera identification and verified its vulnerability to Fast Gradient Sign Method (FGSM) [103] and Jacobian-based Saliency Map Approach (JSMA) [104] attacks. Marra et al. [29] evaluated the vulnerability of deep learning-based source camera identification algorithms to adversarial attacks in a more comprehensive manner. In this work, four kinds of deep learning-based methods (shallow CNN, DenseNet-40, DenseNet-121, XceptionNet) and two schemes of adversarial attacks (FGSM, Projected Gradient Descent (PGD) [105]) are tested to study their behaviour when classifying both pixel patches and full resolution images. According to the study, deep learning-based approaches are vulnerable to adversarial attacks. The authors report that, even if the robustness of deep learning-based methods can be improved by using adversarial training or training with JPEG-compressed images, the resulting networks are still vulnerable to targeted attacks. Zhao et al. [106] generated increasingly strong adversarial examples by using FGSM and Least-likely Class Method (LLCM) [107] and verified the vulnerability of deep learning-based image forensic algorithms. Fan et al. [108] proposed two kinds of gradient-based attacks against deep learning-based recaptured image forensic schemes: single attack and multiple attack. In order to verify the effectiveness of the proposed approach, the authors performed the attack on deep learning-based methods which employed adversarial training (as proposed by Szegedy et al. [109]) as a defense. The results indicated that single attack is ineffective on models that employ adversarial training, while multiple attack with a slow learning rate will obtain better results. Besides generating adversarial examples, generative adversarial networks attracted extensive attention because of their ability to generate photorealistic pictures and to achieve image-to-image translations. Two kinds of attacks against CNN models for source forensics have been proposed by modifying the GAN framework. Focusing on source camera identification, Chen et al. [110] defined a new loss function for the generator comprised of three terms: the perceptional loss, the classification loss, and the adversarial loss. More specifically, the perceptional loss describes the mean absolute difference between the original image and its falsified copy. The classification loss was designed to measure the difference between the output of the camera model identification classifier for the falsified image and the ideal output for the target camera model. The adversarial loss represents the standard loss function of GANs. In order to fool deep learning-based methods for recaptured image detection, Zhao et al. [111] proposed a Cycle-GAN-based scheme by fusing the adversarial loss, the cycle consistency loss and the low frequency consistency loss. In addition to the loss function used in Cycle-GAN, a low frequency consistency loss based on a median filter is proposed to keep the generated image similar to the original one.

Counter anti-forensics [112] methods have been proposed as a defense against anti-forensics techniques by improving the robustness of image forensics methods in case of anti-forensics attacks. Meanwhile, with the advent of adversarial examples, numerous approaches have been developed in the field of computer vision to defend against adversarial attacks [113,114]. According to the review paper on the threat of adversarial attacks by Akhtar and Mian [113], until now defenses against adversarial attacks can be broadly divided into three categories. The first kind of defense is based on using modified examples either during training or during test. Adversarial training is based on this idea. The second kind resorts to modifying the networks by adding more layers, changing loss and so on. The last kind employs external models as network add-on when classifying unseen examples. It should be mentioned that deep learning and adversarial attacks greatly contributed to the cooperation between computer vision and image forensics communities, thus accelerating the development of related image forensics techniques. We will now introduce three preliminary works proposed by source image forensics researchers. Zhao et al. [106] combined adversarial training and regularization of input gradients as a defense against FGSM and LLCM attacks. Firstly, the regularization term of input gradients is added into the original loss function; then, the adversarial training strategy is applied to train the CNN model. Carrara et al. [115,116] used OverFeat, a well-known and successful deep convolutional network architecture, for the image representations. The features were extracted from the pool5 layer of the OverFeat and fed into a k-nearest neighbors (KNN) regressor to get a score. If the score is below threshold, the query image will be classified as an adversarial example and thrown away. From the perspective of image forensics, Schöttle et al. [117] presented an adversarial example detection scheme based on simple steganalysis features. Images that are not classified as adversarial examples will be distinguished by the CNN model [105]. It should be noted that those three approaches were only tested on some relatively old attack methods, such as FGSM, LLCM, FGS, PGD, which are easy to defend against according to the report by Carlini and Wagner [118].

More effective attack algorithms have been proposed in the field of computer vision; it is thus necessary to further study those methods in order to verify their performance in case of more powerful attacks. Thanks to the effort of Papernot et al. [119] a Python library which implements sixteen different kinds of adversarial attacks is available for other researchers to test their proposed defense schemes.

## 8. Evaluation Measures and Datasets

In this section we will describe the evaluation protocol and the datasets used in the reviewed papers.

The metric used in most of the papers is the accuracy:

(3) $$Acc=\frac{TP+TN}{TP+FP+TN+FN}$$

where TP,TN mean the number of correctly classified positive and negative cases, FP,FN represent the number of incorrectly classified positive and negative cases, respectively.

Besides, in order to fairly evaluate the performance for original and manipulated images in case of unbalanced datasets, a weighted accuracy is also used in some papers:

(4) $$Acc_{\mathrm{weighted}}=\frac{7}{10}Acc_{\mathrm{unaltered}}+\frac{3}{10}Acc_{\mathrm{altered}}$$

where $Acc_{\mathrm{unaltered}},Acc_{\mathrm{altered}}$ denote the accuracies in the case of unaltered images dataset and altered images dataset, respectively.

We will now introduce the publicly available image forensics datasets used for evaluating performance as shown in Table 3 and Table 4. Datasets are organized according to the forensics topics. As some anti-forensics and counter anti-forensics methods use datasets introduced in the other sections, we will only report them once. Some of the datasets are freely downloadable from the authors’ websites, while others can be obtained upon request.

### 8.1. Source Camera Identification

Dresden Image Dataset [62] was built for the purpose of developing and benchmarking camera-based digital forensic techniques. There are more than 14,000 images captured by 73 devices which belong to 25 different models. Dresden dataset was released in 2010, when smartphones were not yet a popular way to take pictures and therefore this dataset does not contain pictures taken with such devices.

VISION [64] is a video and image dataset for source identification. It is currently composed by 34,427 images and 1914 videos, both in the native format and in their social version (Facebook, YouTube, and WhatsApp are considered), from 35 modern smartphones/tablets of 11 major brands.

SPC2018 [7] is a dataset published in 2018 for the IEEE Signal Processing Cup competition whose topic was camera model identification. This dataset consists of 2750 images from ten different camera models (including point-and-shoot cameras, cell phone cameras, and digital single-lens reflex cameras), with 275 images captured using each camera model.

### 8.2. Recaptured Image Forensic

LS-D [53] is a large-scale dataset for evaluating recaptured image forensic. Four kinds of recapture attacks are considered: (1) photographing a printed copy of an image; (2) scanning a printed copy of an image; (3) photographing a displayed image; and (4) capturing a screen-grab of displayed image. This dataset consists of 145,000 pairs of original and recaptured images. A diverse set of devices has been used to recapture the images: 234 displays, 173 scanners, 282 printers, and 180 recaptured cameras.

NTU-Rose [65] is a collection of images recaptured from LCDs with good quality. It is composed of 2700 recaptured images captured by using three digital still cameras and three LCDs. The number of the original images is 300, including 100 images taken by the three cameras, 100 images downloaded from Flick, and 100 tampered images.

ICL [68] is a dataset of images recaptured from a LCD and consists of 1035 original images taken by nine different cameras and 2520 images recaptured by using different devices. Camera settings were tuned in order to maintain a high image quality. Therefore, this dataset provides high-quality, high-resolution recaptured images.

ASTAR [67] is a smartphone images dataset for single image recaptured detection. The dataset is divided in three subsets. Subset A consists of 1094 real-scene images and 1137 recaptured images with real environment background. Subset B is built by cropping the real-scene images from Subset A, and by adding 1765 recaptured images without real environment backgrounds. Subset C consists of 587 pairs of single captured and recaptured images through transforming and cropping.

### 8.3. CG Image Detection

Columbia [69] consists of 800 CG images downloaded from Internet, 1200 images from personal collections, 800 original images from Google Image searches, and 800 recaptured CG images.

DSTok [71] includes CG and real photograph (PG) images collected from the Internet. There are 4850 pairs of CG and PG images. All of them were JPEG-compressed and the file sizes were between 12 KB and 1.8 MB.

WIFS [58] is built for new CG and PG images. There are 1800 CG images and 1800 PG images. CG images were downloaded from the Level Design Reference Database, which contains more than 60,000 good resolution video-game screenshots in JPEG format. Only five different video games were judged photo realistic enough to be included in WIFS, and thus only 1800 images were selected. PG images are high-resolution images taken from the RAISE dataset and directly converted to JPEG format.

3Dlink [55] consists of 6800 CG images download from the 3Dlink website and 6800 PG images captured under various environmental conditions by using different camera models.

### 8.4. GAN-Generated Image Detection

MFS2018 [6] is a dataset released in 2018 for a media forensics challenge, which aims to help advance the state-of-the-art for image and video forensics techniques. For what concerns the works reviewed in this paper, only a subset of the dataset was used (GAN Crop and GAN Full).

### 8.5. Social Network Identification

UCID social [75] is a UCID-based dataset for social network identification. Images from UCID were first JPEG-compressed with different quality factors. Then, those compressed images were uploaded to and subsequently downloaded from Flickr, Facebook, and Twitter. The UCID social dataset is composed of 30,000 images, 10,000 images from each social network.

The PUBLIC social dataset [75] consists of 3000 uncontrolled images with different sizes, JPEG quality factors and contents. Those images were directly downloaded from different social networks, including Flickr, Facebook, and Twitter, 1000 images for each one.

IPLAB [76] provides 2720 images in JPEG format. Ten social networks were considered for this dataset: Facebook, Google+, Twitter, Flickr, Instagram, Tumblr, Imgur, Tinypic, Whatsapp, and Telegram. Captured images were uploaded to the social networks either by using a web browser, or by using iOS and Android native apps.

## 9. Discussion and Conclusions

In this paper we presented a comprehensive survey of deep learning-based source image forensics, anti-forensics, and counter anti-forensics. According to our review, deep learning-based contributions for source image forensics can be divided into five categories, as shown in Figure 2: adopting traditional convolutional neural networks, or improving performance adopting strategies such as data enhancement, fusion and ensemble, patch selection, or using different classifiers. Most researchers based their methods on popular CNNs for computer vision, such as ResNet, XceptionNet, and DenseNet. Those architectures have proven to be effective when dealing with source image forensics tasks, even though they were originally developed for different scenarios. In particular, the most popular architecture for source image forensics appears to be ResNet, which strikes a balance between computational complexity and performance. As deep learning methods are data-driven, many contributions are focused on data enhancement techniques to improve the signal-to-noise ratio of data provided to CNNs. To do so, both separate pre-processing steps and customized network layers have been proposed. While many works reported an improvement in networks performance by using these methods, some authors obtained better results without using them. Unfortunately, these inconsistencies in different works are currently unavoidable: every method uses a different experimental protocol, thus making it impossible to compare the results. Moreover, as deep learning methods are heavily dependent on training data, it is difficult to declare a winner when comparing methods that have been evaluated on different datasets.

In conclusion, while many deep learning-based source image forensics methods have obtained remarkable results, there are still many research opportunities in this field worthy of being explored. Interested researchers may draw inspiration from the ever-expanding set of machine learning architectures and techniques to build new methods. Finally, it would be very important for the advancement of this research area to come up with a standard experimental protocol and shared datasets to make it possible to fairly compare the different proposed solutions.

## Figures and Tables

**Table 1:** Summary of the reviewed architectures for source camera identification. CC and IC denote Constrained and Instant convolutional operations, respectively. GAP denotes the presence of a global average pooling operation. BN denotes the presence of batch normalization operations.

Architecture	Input Size	Preprocessing	Convolutional Part					Fully Connected Part		
			N Layers	Activation	Pooling	BN	GAP	N Layers	Activation	Dropout
A1 [23]	48 × 48 × 3	-	3	ReLU	Max	-	-	1	ReLU	✓
A2 [24]	32 × 32 × 3	-	2	L-ReLU	Max	-	-	2	L-ReLU	✓
A3 [25]	36 × 36 × 3	-	3	ReLU	Avg	✓	-	1	ReLU	✓
A4 [26]	64 × 64 × 3	-	13	ReLU	Max	-	-	2	?	✓
A5 [27]	256 × 256 × 3	-	1 Conv, 12 Residual	ReLU	-	-	✓	-	-	-
A6 [36]	64 × 64 × 3	-	4	?	Max	-	-	1	ReLU	-
A7 [37]	64 × 64 × 3	-	10	?	Max	-	-	1	ReLU	-
A8 [38]	256 × 256 × 2	IC + CC	4	TanH	Max, Avg	✓	-	2	TanH	-
A9 [39]	256 × 256	HP	3	ReLU	Max	-	-	2	ReLU	✓
A10 [41]	256 × 256 × 3	LBP	3	ReLU	Max	✓	-	2	ReLU	✓
A11 [42]	64 × 64 × 3	-	6	ReLU	Avg	✓	✓	-	-	-
A12 [43]	64 × 64 × 3	-	1 Conv, 3 Residual	ReLU	Avg	-	✓	-	-	-

**Table 2:** Summary of the reviewed architectures for recaptured image forensics (RF), CG image detection (CGI), GAN-generated images detection (GAN), and Source social networks identification (SSN). Lap denotes Laplacian filter, GR denotes the Guassian residuals. Col + Tex is the combination of Cb,Cr and the texture filtering responses by Schmidt filter bank. Filters denotes a combination of three kinds of high-pass filters used for steganalysis and DCT-His denotes the histogram of DCT coefficents.

Architecture		Input size	Preprocessing	Convolutional part					Fully connected part		
				N Layers	Activation	Pooling	BN	GAP / Stats	N Layers	Activation	Dropout
RF	B1 [50]	N × N × 3	Lap	5	ReLU	Avg	✓	GAP	-	-	-
	B2 [53]	64 × 64 × 1	GR	6	L-ReLU	-	✓	-	1	L-ReLU	-
	B3 [52]	32 × 32 × 3	Conv	2	ReLU	Avg	✓	-	1	?	-
	B4 [51]	64 × 64 × 3	-	6	ReLU	Max	-	-	2	ReLU	✓
CGI	C1 [54]	32 × 32 × 3	-	6	ReLU	-	-	-	2	ReLU + BN	-
	C2 [55]	96 × 96	Col + Tex	4	ReLU	Avg	✓	-	1	?	✓
	C3 [56]	650 × 650	Filters	5	ReLU	Avg	✓	GAP	-	-	-
	C4 [57]	NxN	Conv	3	ReLU	Max	✓	-	1	ReLU	✓
	C5 [58]	100 × 100 × 1	-	2	-	-	-	Stats	1	ReLU	✓
GAN	D1 [59]	N × N × 3	Lap	3	L-ReLU	Max	-	-	2	L-ReLU	-
SSN	E1 [60]	64 × 64	DCT-His	2	ReLU	Max	-	-	1	ReLU	✓
	E2 [61]	64 × 64	PRNU	4	ReLU	Max	-	-	1	ReLU	✓

**Table 3:** The experimental setting for different algorithms. In this table, DA, FE, PS, C denote respectively data augmentation, fusion and ensemble, patch selection, and classifiers. The ratio between training and test data is shown in the column “Train: Test”. For the performance-patch/voting, the numbers between parenthesis denote the number of models/sensors in the test set. It should be noted that the evaluation matrix for 8, 20 is that 0.7 × (accuracy of unaltered images) + 0.3 × (accuacy of manipulated images). Some works evaluated the performance on multiple datasets; only the most representative ones are shown in this table.

	Arch.	Input Size	D.A.	F./E.	P.S.	C.	Train: Test	Dataset	Perf. (Patch)		Perf. (Voting)	
									Model	Sensor	Model	Sensor
[23]	A1	48 × 48 × 3	-	-	-	Softmax	7:3	Dresden [62]	72.9% (27)	29.8% (74)	94.1% (27)	-
[24]	A2	32 × 32 × 3	-	-	-	Softmax		MICHE-I [63]	98.1% (3)	91.1% (5)	-	-
[25]	A3	36 × 36 × 3	-	-	-	SVM	8:2	Dresden [62]	-	-	-	99.9% (10)
[26]	A4	64 × 64 × 3	-	-	✓	Softmax	3:2	Dresden [62]	93% (25)	-	>98% (25)	-
[27]	A5	256 × 256 × 3	-	-	-	Softmax	7:3	Dresden [62]	94.7% (27)	45.8% (74)	-	-
[29]	A6	64 × 64 × 3	-	-	-	Softmax	8:2	VISION [64]	-	80.77% (35)	-	97.47% (35)
	DenseNet-40	32 × 32 × 3							-	87.96% (35)	-	95.06% (35)
	DenseNet-121	224 × 224 × 3							-	93.88% (35)	-	99.10% (35)
	XceptionNet	299 × 299 × 3							-	95.15% (35)	-	99.31% (35)
[31]	DenseNet-201 + SE-Block	256 × 256 × 1	✓	✓	✓	SE-block	3.2:1	SPC2018 [7]	98.37% (10, weighted)	-	-	-
[36]	A6	64 × 64 × 3	-	-	✓	SVM		Dresden [62]	93% (18)	-	>95 % (18)	-
[37]	A7	64 × 64 × 3	-	-	✓	Softmax		Dresden [62]	94.93% (18)	-	-	-
[38]	A8	256 × 256 × 2	✓	✓	-	ET	4:1	Dresden [62]	98.58% (26)	-	-	-
[39]	A9	256 × 256	-	-	-	Softmax	8:2	Dresden [62]	98.99% (12) 98.01% (14)	-	-	-
[41]	A10	256 × 256 × 3	✓	-	-	Softmax	8:2	Dresden [62]	98.78% (12) 97.41% (14)	-	-	-
[43]	A12	64 × 64 × 3	✓	✓	✓	Softmax	4:1	Dresden [62]	-	97.03% (9)	-	-
[32]	DenseNet-161	480 × 480 × 3	✓	-	-	Softmax		SPC2018 [7]	98% (10, weighted)	-	-	-
[42]	A11	64 × 64 × 3	✓	✓	-	Softmax	4:1	Dresden [62]	-	94.14% (9)	-	-
[33]	Inception-Xception	299 × 299	-	✓	✓	Softmax		SPC2018 [7]	93.29% (10, weighted)	-	-	-
[28]	ResNet-modified	48 × 48 × 3	✓	-	-	Softmax		Dresden [62]	-	-	79.71% (27)	53.4% (74)

**Table 4:** The experimental setting for different algorithms for recaptured image forensics (RF), CG image detection (CGI), and Source social networks identification (SSN). In this table, DA, FE, PS, C denote respectively data augmentation, fusion and ensemble, patch selection, and classifiers. The ratio between training and test data is shown in the column “Train: Test”. For the performance-patch/voting, the numbers between parenthesis denote the patch sizes, when applicable.

		Arch.	Input Size	D.A.	F./E.	P.S.	C.	Train: Test	Dataset	Perf. (Patch)	Perf. (Voting)
RF	[50]	B1	N × N × 3	✓	-	-	Softmax	1:1	NTU-Rose [65] LCD_R [66]	99.74% (512) 99.30% (256) 98.48% (128) 95.23% (64)	
	[53]	B2	64 × 64 × 1	✓	✓	-	Softmax	8:2	LS-D [53]	99.90%	
	[52]	B3	32 × 32 × 3	✓	-	-	Softmax	1:1	ASTAR [67]	86.78%	93.29% (64)
									NTU-Rose [65]	96.93%	98.67% (64)
									ICL [68]	97.79%	99.54% (64)
	[51]	B4	64 × 64 × 3	-	-	-	Softmax	1:1	ICL [68]	85.73%	96.60%
CGI	[54]	C1	32 × 32 × 3	-	-	-	Softmax	3:1	Columbia [69]		98%
	[70]	ResNet50	224 × 224	-	-	-	Softmax	5-f CV	DSTok [71]	96.1%	
	[55]	C2	96 × 96	✓	✓	-	Softmax	13:4	3Dlink [55]	90.79%	94.87% (192)
	[56]	C3	650 × 650	✓	-	-	Softmax	9:8	WIFS [58]	99.95%	100%
	[72]	ResNet50	?	✓	-	-	Softmax	7:1	Columbia [69]	98%	
	[57]	C4	233 × 233	✓	-	✓	Softmax	3:1	Columbia [69]	85.15%	93.20%
	[58]	C5	100 × 100 × 1	-	✓	-	MLP	8:2	WIFS [58]	84.80%	93.20%
	[73]	VGG19		-	✓	✓	MLP	5:2	WIFS [58]	96.55%	99.89%
	[74]	ResNet50	224 × 224 × 3	-	-	-	SVM		DSTok [71]	94%	
SSN	[60]	E1	64 × 64	✓	-	-	Softmax	9:1	UCID [75]	98.41%	95% (Avg.)
									PUBLIC [75]	87.60%	
									IPLAB [76]	90.89%	
	[61]	E2	64 × 64	✓	-	-	Softmax	9:1	UCID [75]	79.49%	90.83%
									VISION [64]	98.50%	
									IPLAB [76]		83.85%

**Table 5:** The statistical table for GAN-generated image detection. GAN shows the GAN model used to generate the images. Method represents the detection algorithm. Performance is the obtained accuracy unless otherwise specified. Only the best performance described in each paper is reported in this table.

	GAN	Dataset	Method	Performance
[82]	Cycle-GAN [87]	Cycle-GAN Data [87]	Cycle-GAN Discriminator [87]	83.58%
			Fridrich and Kodovsky [83]	94.40%
			Cozzolino et al. [84]	95.07%
			Bayar and Stamm [85]	84.86%
			Rahmouni et al. [58]	85.71%
			DenseNet [18]	89.19%
			InceptionNet V3 [86]	89.09%
			XceptionNet [19]	94.49%
[88]	DC-GAN W-GAN	CelebA [92]	DCGAN Discriminator	95.51%
			VGG+FLD	>90 % (DC-GAN) >94% (W-GAN)
[91]	DFC-VAE DCGAN WGAN-GP PGGAN	CelebAHQ [93] CelebA [92] LFW [94]	Co-Color	100%
[59]	PG-GAN	CelebAHQ [93]	Lap-CNN	96.3%
[98]	GAN	MFS2018 [6]	RG-INHNet	0.56 (AUC)
			Saturation Features	0.7 (AUC)
[100]	Cycle-GAN Pro-GAN Star-GAN	MFS2018 [6]	PRNU-based method	0.999 (AUC)
